# Treating Intractable Nausea and Vomiting in Cerebellar and Medullary Stroke

**DOI:** 10.7759/cureus.114288

**Published:** 2026-08-10

**Authors:** Adil Bhatti, Peter Schultz, Shantanu Amin, Taimur Khalid, John Varras

**Affiliations:** 1 College of Osteopathic Medicine, Kansas City University, Joplin, USA; 2 College of Osteopathic Medicine, Kansas City University, Kansas City, USA; 3 College of Medicine, Kansas City University, Joplin, USA; 4 Department of Internal Medicine, Kirk Kerkorian School of Medicine at University of Nevada, Las Vegas, Las Vegas, USA

**Keywords:** antiemetic therapy, area postrema, cerebellar stroke, intractable nausea, medullary stroke, posterior circulation stroke, vomiting

## Abstract

Cerebellar and medullary stroke can cause persistent nausea and vomiting that may be resistant to initial antiemetic monotherapy and interfere with oral intake and recovery. Evidence guiding management remains limited. We report a 48-year-old man with diabetes mellitus and acute-to-subacute infarcts involving the inferomedial right cerebellum and posterior right medulla who presented with 10 days of persistent nausea and vomiting accompanied by focal neurologic symptoms. Diabetic gastroparesis was considered, but abdominal imaging and gastric emptying scintigraphy did not support a contributory gastrointestinal cause. Ondansetron and metoclopramide provided only limited or temporary relief. Symptoms improved after sequential escalation to ondansetron, chlorpromazine, prochlorperazine, and amitriptyline; transdermal scopolamine was included in the discharge regimen. Nausea and vomiting were nearly resolved before discharge and fully resolved by three-month follow-up. Because multiple medications were introduced over a short interval and natural neurologic recovery may have contributed, the effect of any individual agent or combination cannot be determined. This case suggests that receptor-diverse pharmacotherapy may be considered when initial treatment fails while underscoring the need for further study of efficacy, safety, and medication sequencing.

## Introduction

Cerebellar and medullary strokes comprise approximately 1%-7% of ischemic infarcts [1,2] and can cause substantial morbidity, functional limitation, and adverse outcomes [3-6]. Nausea and vomiting occur frequently in posterior circulation stroke [4]. Cerebellar infarction can disrupt the integration of vestibular signals from the inner ear and brainstem, producing dizziness, imbalance, nausea, and vomiting [7]. Medullary infarction may involve the area postrema, a structure in the dorsal medulla that is directly exposed to circulating substances because it lacks a typical blood-brain barrier [8,9]. Injury to this region can produce persistent nausea, vomiting, and hiccups that may be mistaken for primary gastrointestinal disease [8,9]. When refractory, these symptoms can impair oral intake and hydration, complicate participation in rehabilitation, and delay discharge [8,9].

Standard antiemetics act through different pharmacologic pathways. Ondansetron is a selective serotonin 5-hydroxytryptamine-3 receptor antagonist, whereas metoclopramide is a dopamine D2-receptor antagonist with prokinetic activity [10]. However, incomplete responses to these agents have been described in stroke-associated nausea and vomiting [8,9]. Published evidence is largely limited to case reports, and, to our knowledge, no established treatment guideline or standardized medication sequence exists for this complication. We report a patient with cerebellar and posterior medullary infarction whose refractory nausea and vomiting improved after sequential escalation to receptor-diverse pharmacotherapy.

## Case presentation

**Figure 1:**
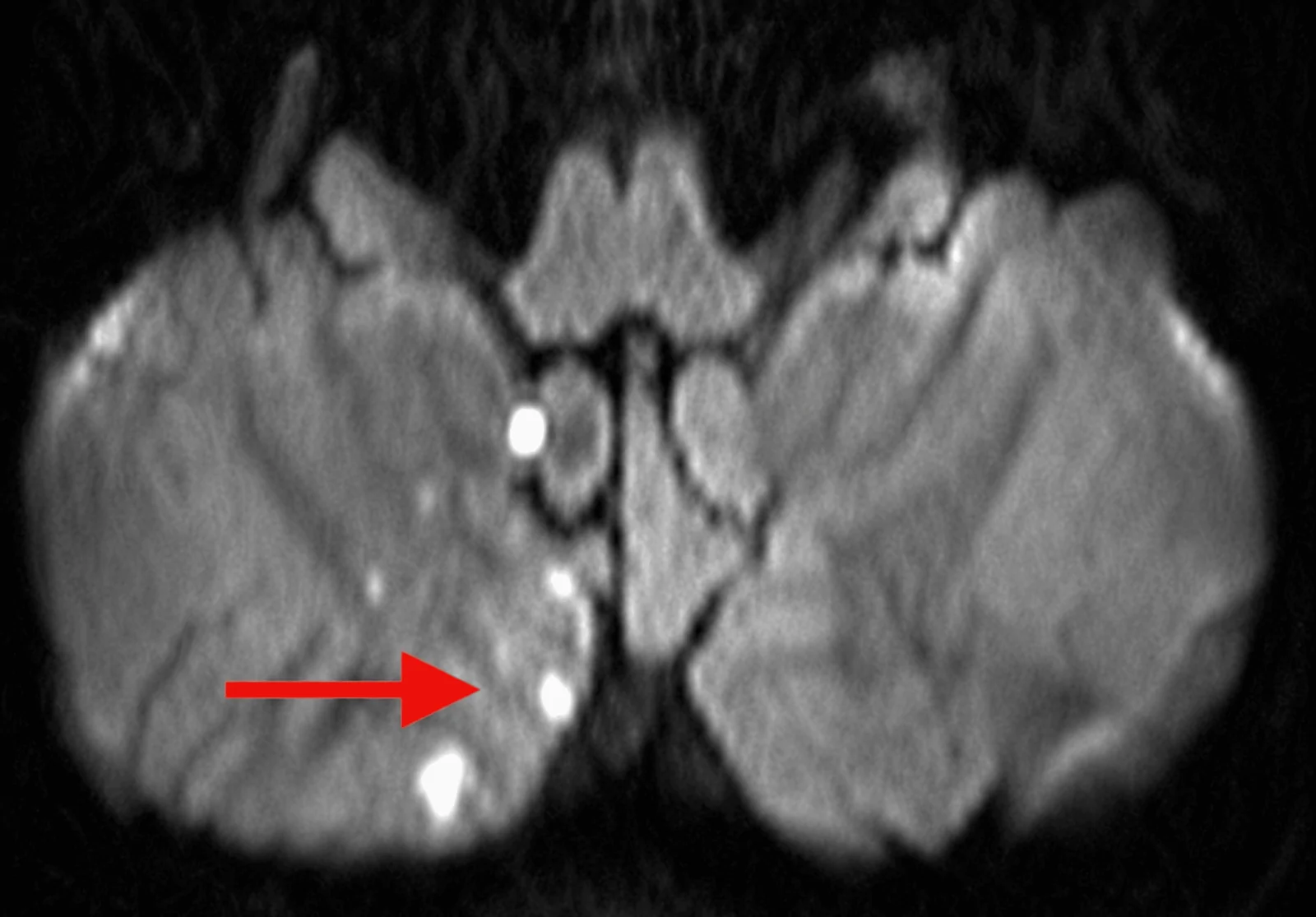
Diffusion-weighted magnetic resonance imaging demonstrating right cerebellar infarcts Axial diffusion-weighted magnetic resonance imaging demonstrates multiple hyperintense foci in the inferomedial right cerebellar hemisphere, consistent with acute-to-subacute infarcts. The red arrow indicates a representative focus. A separate acute-to-subacute infarct in the posterior right medulla was identified on another image from the examination and is not shown in this figure

A 48-year-old man with type 2 diabetes mellitus presented to the emergency department with 10 days of persistent nausea, vomiting, and right-sided weakness with paresthesias. Vital signs were stable, and neurologic examination showed mild right-sided weakness and sensory loss without cranial nerve deficits. Computed tomography imaging of the brain without contrast revealed no acute hemorrhage. The patient had previously been hospitalized at another facility for a stroke and discharged after five days but did not obtain the prescribed medications. Brain magnetic resonance imaging (MRI) performed at the outside facility was reviewed and showed acute-to-subacute infarcts in the inferomedial right cerebellum and posterior right medulla. Repeat brain MRI reaffirmed these findings along with a few new infarcts visible in the same region (Figure 1). He was started on aspirin and a high-intensity statin.

Upon admission, the patient was kept nil per os for aspiration precautions and received intravenous fluids. Ondansetron was ordered as needed and provided only mild initial relief, with worsening symptoms the following day. Examination demonstrated right-sided dysdiadochokinesia and finger-to-nose dysmetria. Strength was 5/5 in the bilateral lower extremities and left upper extremity and 4/5 in the right upper extremity. Magnetic resonance angiography of the neck also demonstrated approximately 50% stenosis of the proximal left internal carotid artery; this anterior-circulation finding did not anatomically correspond to the right cerebellar and posterior medullary infarcts. Admission blood glucose was 302 mg/dL, and hemoglobin A1c was 13.5%. Because poor glycemic control raised concern for diabetic gastroparesis, metoclopramide was added as needed. The patient reported temporary relief, followed by recurrent worsening of nausea, vomiting, and dizziness the following day. Three-view abdominal radiographs showed no contributory abnormality. Gastric emptying scintigraphy demonstrated 74%, 89%, 90%, and 95% gastric emptying at 15, 30, 45, and 60 minutes, respectively, with 5% gastric retention at 60 minutes (Table 1). The study did not support delayed gastric emptying as the primary explanation for the patient’s symptoms.

**Table 1:** Quantitative gastric emptying scintigraphy results Gastric emptying and retention percentages were measured following administration of a Tc-99m-labeled test meal. The interpreting nuclear medicine report characterized the study as not demonstrating delayed gastric emptying. Because the available report did not provide the meal composition or laboratory-specific reference intervals, generic solid-meal reference thresholds were not applied Tc-99m: technetium-99m

Time after meal (minutes)	Gastric emptying (%)	Gastric retention (%)
15	74	26
30	89	11
45	90	10
60	95	5

As the gastric-emptying evaluation did not support delayed gastric emptying as the primary explanation, metoclopramide was discontinued, and the ondansetron dose was increased to 8 mg every eight hours. Despite this change, the patient reported worsening nausea and an inability to tolerate food or liquids. He subsequently developed persistent hiccups. The dysdiadochokinesia and finger-to-nose dysmetria were consistent with cerebellar dysfunction, while the new persistent hiccups in the setting of a posterior medullary infarct raised concern for dorsal medullary and area postrema involvement. Given the focal neurologic findings, MRI-confirmed cerebellar and posterior medullary infarcts, associated dizziness, and gastrointestinal evaluation that did not identify a primary cause, a central neurologic etiology for the nausea and vomiting was favored.

Because symptoms persisted, treatment was escalated. Chlorpromazine 25 mg every four hours was added for persistent hiccups, and prochlorperazine 10 mg every six hours was added for refractory nausea. Gastroenterology was consulted, and esophagogastroduodenoscopy demonstrated grade C esophagitis with linear ulcerations considered secondary to repeated vomiting. After ondansetron, chlorpromazine, and prochlorperazine were administered together, the treating team documented a marked reduction in emesis and resumption of oral intake, although the hiccups persisted. Amitriptyline 25 mg nightly was then added, and the patient reported near-resolution of nausea by the following night.

At discharge, the medication regimen consisted of ondansetron 8 mg every eight hours, prochlorperazine 10 mg every six hours, amitriptyline 25 mg nightly, and transdermal scopolamine replaced every three days. At one-week follow-up, the patient reported near-complete resolution of nausea and vomiting, no recurrence of hiccups, and only mild residual paresthesias. At three-month follow-up, he reported intermittent tunnel vision and residual right-sided weakness and paresthesias but remained ambulatory. His nausea and vomiting had fully resolved. The documented sequence of symptoms, treatment changes, principal pharmacologic targets, and clinical responses is summarized in Table 2.

**Table 2:** Sequence of clinical findings, pharmacologic interventions, and documented responses Exact calendar dates, medication start and stop times, the precise duration of NPO status, validated standardized quantitative nausea ratings, daily emesis counts, quantitative oral-intake measurements, and the precise interval between each medication change and clinical response were not available in the accessible retrospective records. The table reflects the documented relative sequence of treatment changes and clinical responses. Principal antiemetic classes and mechanisms for ondansetron, metoclopramide, chlorpromazine, prochlorperazine, and scopolamine are summarized from Athavale et al. [10]. The contribution of any individual medication cannot be determined NPO: nil per os; 5-HT3: 5-hydroxytryptamine-3

Relative clinical stage	Clinical findings and working interpretation	Intervention	Principal pharmacologic target or class	Documented response
Presentation and early admission	Ten days of nausea and vomiting with right-sided neurologic symptoms; cerebellar and posterior medullary infarcts identified	NPO status, intravenous fluids, and ondansetron as needed	Selective 5-HT3 receptor antagonist	Mild initial relief followed by worsening symptoms the next day
Initial gastrointestinal-directed treatment	Poor glycemic control raised concern for diabetic gastroparesis	Metoclopramide as needed	Dopamine D2-receptor antagonist with prokinetic activity	Patient reported temporary relief followed by recurrent nausea, vomiting, and dizziness
After gastric evaluation	Gastric-emptying and abdominal imaging did not identify delayed gastric emptying or another contributory gastrointestinal abnormality	Metoclopramide discontinued; ondansetron increased to 8 mg every 8 hours	Intensified 5-HT3 receptor antagonism	Continued worsening, inability to tolerate food or liquids, and development of persistent hiccups
Multimodal inpatient escalation	Central neurologic etiology favored; persistent nausea, vomiting, and hiccups	Chlorpromazine 25 mg every four hours and prochlorperazine 10 mg every six hours added	Phenothiazine antiemetics with prominent dopamine D2-receptor antagonism	Treating team documented marked reduction in emesis and resumption of oral intake; hiccups persisted
Subsequent inpatient escalation	Residual nausea and persistent hiccups	Amitriptyline 25 mg nightly added	Tricyclic neuromodulator with serotonergic, noradrenergic, antihistaminic, and antimuscarinic activity; exact antiemetic contribution uncertain	Patient reported near-resolution of nausea by the following night
Discharge and one-week follow-up	Substantial improvement before discharge	Continued ondansetron, prochlorperazine, and amitriptyline; transdermal scopolamine documented in the discharge regimen	Scopolamine provides muscarinic receptor antagonism and acts on vestibular pathways	Near-complete resolution of nausea and vomiting and no recurrent hiccups at one week
Three-month follow-up	Residual neurologic symptoms but no recurrent gastrointestinal symptoms	Clinical follow-up	Not applicable	Complete resolution of nausea and vomiting

## Discussion

Nausea and vomiting are prevalent and potentially debilitating manifestations of cerebellar and medullary infarction, arising from disruption of vestibular pathways or direct involvement of the area postrema. Cerebellar injury can impair the integration of vestibular signals, producing dizziness, imbalance, nausea, and vomiting [7]. Dorsomedial medullary infarction may involve the area postrema and produce persistent nausea, vomiting, and hiccups [8,9].

In this patient, ondansetron and metoclopramide provided only limited or temporary relief. Cohen et al. reported relief of ischemic area postrema syndrome after treatment with metoclopramide 10 mg four times daily and ondansetron 8 mg every eight hours [9]. This report informed the decision to increase ondansetron in our patient, although his symptoms persisted. Na et al. similarly described persistent nausea, vomiting, and poor oral intake after medullary infarction that did not respond to metoclopramide and ondansetron and improved only after additional prokinetic therapy [8]. These differing responses illustrate the variability of stroke-associated central emesis. In the present case, clinical improvement occurred after chlorpromazine and prochlorperazine were added, with further improvement after amitriptyline was introduced; however, the contribution of any individual medication or combination cannot be determined.

Other reports of neurologically mediated vomiting have described the use of fosphenytoin and levetiracetam for ictal vomiting after cerebellar hemorrhage [11] and low-dose olanzapine for central vertigo [12]. Although these clinical contexts differ from ischemic area postrema syndrome, they illustrate the use of mechanism-directed treatment for refractory central nausea and vomiting. Reports of medullary infarction involving or adjacent to the area postrema also demonstrate variable responses to conventional antiemetics [8,9,13]. Taken together, the limited literature supports individualized, stepwise treatment rather than a standardized multidrug regimen.

Although combining medications that act through different pathways may broaden antiemetic coverage, it also creates overlapping safety concerns. Ondansetron and dopamine-antagonist antiemetics may affect cardiac conduction, while phenothiazines such as chlorpromazine and prochlorperazine may also cause sedation, hypotension, and extrapyramidal reactions. Scopolamine contributes anticholinergic effects, and the inclusion of amitriptyline adds another centrally acting and potentially sedating medication. Multidrug regimens should therefore be individualized, with attention to hemodynamic status, mental status, extrapyramidal symptoms, anticholinergic effects, concurrent medications, and electrocardiographic findings [10].

This report has several limitations. It describes a single patient and is based on retrospective review of the available clinical documentation. Exact medication start and stop times, the precise duration of nil per os status, validated standardized quantitative nausea ratings, daily emesis counts, quantitative oral-intake measurements, and detailed serial vestibular, gait, swallowing, and cranial nerve examinations were not available. Because medications were added sequentially and sometimes concurrently, the contribution of any individual agent or combination cannot be isolated, and natural neurologic recovery may have contributed to symptom improvement. Poor glycemic control was a potential confounder; only the admission glucose and hemoglobin A1c were available for analysis, and serial glycemic trends could not be assessed. Complete corrected QT data, blood-pressure trends, and systematic adverse-effect assessments were also not available in the accessible record. Therefore, the observed temporal association between treatment escalation and clinical improvement should be considered hypothesis-generating rather than evidence of the efficacy or safety of a specific multidrug regimen.

## Conclusions

Persistent nausea, vomiting, and hiccups may accompany cerebellar and medullary infarction and may be resistant to initial antiemetic monotherapy. In this patient, symptoms improved after sequential escalation to medications acting through several pharmacologic pathways. Because multiple agents were introduced over a short interval and natural neurologic recovery may have contributed, this case cannot establish causation, safety, or the optimal medication combination. Receptor-diverse pharmacotherapy may be considered on an individualized basis when initial treatment is unsuccessful, but further study is needed to determine efficacy, safety, and optimal medication selection and sequencing.
